# Complete genome sequences of three biocalcifying bacterial strains, *Lysinibacillus fusiformis* LBL1, *Peribacillus frigoritolerans* PBL6, and *Planococcus glaciei* PCL1, isolated from Lebanese calcareous soil

**DOI:** 10.1128/mra.00818-26

**Published:** 2026-10-14

**Authors:** Shirine Kyprianos, Nancy Fayad, Rafik Belarbi, Ameur El Amine Hamami, Jad Abdallah

**Affiliations:** 1 Multi-Omics Laboratory, School of Pharmacy, Lebanese American University 37607, Byblos, Lebanon; 2 Laboratoire des Sciences de l’Ingénieur pour l’Environnement (LaSIE), UMR CNRS 7356, La Rochelle University 131927, La Rochelle, France; 3 Department of Architecture, Canadian University Dubai, Dubai, United Arab Emirates; 4 Department of Civil and Building Engineering, Université de Sherbrooke 7321, Sherbrooke, Canada; California State University San Marcos, San Marcos, California, USA

**Keywords:** whole genome sequencing, PacBio HiFi sequencing, biocalcification, microbially induced calcium carbonate precipitation (MICP), *Lysinibacillus fusiformis*, *Peribacillus frigoritolerans*, *Planococcus glaciei*, urease, carbonic anhydrase

## Abstract

We report the complete genome sequences of three biocalcifying bacteria, *Lysinibacillus fusiformis* LBL1, *Peribacillus frigoritolerans* PBL6, and *Planococcus glaciei* PCL1, isolated from calcareous soil in Laqlouq, Lebanon. With both ureolytic and non-ureolytic phenotypes, this study provides the genomic resources for investigating the diversity of biocalcifying bacteria.

## ANNOUNCEMENT

Microbially induced calcium carbonate precipitation (MICP) has attracted considerable interest for biotechnological applications (1). Calcareous soils represent an underexplored bacterial diversity for ureolytic and non-ureolytic MICP. Here, we report the complete genome sequences of three MICP-competent strains from distinct *Bacillota* lineages, isolated from 100 g of calcareous soil collected at a depth of 10 cm in Laqlouq, Lebanon (34.14019° N, 35.86724° E), in July 2024. Briefly, 10 g of sieved soil was suspended in 90 mL of 0.85% sodium chloride (NaCl) and shaken at 130 rpm for 4 h. Aliquots were streaked onto 2% (wt/vol) LB broth (Condalab, Spain, Cat. No. 1231) supplemented with 1.5% (wt/vol) agar (HiMedia, India, Cat. No. GRM026) and incubated at 30°C for 24 h, as per Khoury et al. (2). All three strains precipitated calcium carbonate in laboratory assays, with only LBL1 displaying a ureolytic phenotype on Christensen’s urea agar (3).

For whole genome sequencing (WGS), strains were cultured in Brain Heart Infusion (BHI) broth at 37°C for 18 h with shaking at 150 rpm. Genomic DNA was extracted using the Wizard Genomic DNA Purification Kit (Promega, USA) following the manufacturer’s instructions. PacBio Sequel II HiFi sequencing (Pacific Biosciences, USA) was performed at Apical Scientific (Malaysia). High-molecular-weight DNA was sheared to 6–20 kb using a g-TUBE (Covaris, USA), and HiFi libraries were prepared using a SMRTbell Prep Kit 3.0 and size-selected on a BluePippin system (Sage Science, USA). Circular consensus sequences (CCS) were generated using SMRT Link software (Pacific Biosciences, USA). *De novo* assembly was performed using Unicycler v0.5.1 in bold bridging mode via the Galaxy platform, excluding contigs shorter than 2,000 bp (4). Assembly quality and completeness were evaluated using QUAST v5.3.0 (5) and Benchmarking Universal Single-Copy Orthologs (BUSCO) v5.8.0 (6), respectively. Circularity was confirmed using Bandage v2022.09 (7). Taxonomic identification based on genomic data was performed using Kraken2 v2.17.1 (8). The strains were therefore named as *Lysinibacillus fusiformis* LBL1, *Peribacillus frigoritolerans* PBL6, and *Planococcus glaciei* PCL1.

Plasmid contigs in PBL6 and PCL1 were identified using PlasFlow v1.1.0 (9) and verified by BLASTn against the NCBI Assembly database. Genomes were annotated by NCBI’s Prokaryotic Genome Annotation Pipeline (PGAP) v6.11 (10), with complementary functional annotation using the RASTtk pipeline via the BV-BRC platform (11). Default parameters were used except where noted above.

The LBL1 genome assembled into a single circular chromosome of *ca*. 5 Mbp. The PBL6 genome resolved into a 5.5 Mbp circular chromosome and one 12 Kb plasmid, pPBL6_12. The PCL1 genome comprised a 4 Mbp circular chromosome and two plasmids, pPCL1_72 (72 Kbp) and pPCL1_9 (9 Kbp). BUSCO completeness scores were 94.4%, 96.0%, and 95.1% for LBL1, PBL6, and PCL1, respectively. Genome data were deposited in NCBI GenBank under Bioproject PRJNA1472847. Detailed genomic features are shown in Table 1.

**TABLE 1:** Genomic features of biocalcifying strains LBL1, PBL6, and PCL1

Strain	Replicon	BioSample/NCBI SRA accession	Accession number	Reads/N50 (bp)	Genome coverage (x)	Size (bp)	Total CDS^*a*^	GC content (%)
*Lysinibacillus fusiformis* LBL1	Chromosome	SAMN60519687/SRX33759951	CM176597.1	153,778/10,695	330	4,988,331	4,752	37.46
*Peribacillus frigoritolerans* PBL6	Chromosome	SAMN60519688/SRX33759952	CM176596.1	133,764/12,084	293	5,506,947	5,241	40.46
	pPBL6_12		JBYTNV010000002.1			12,121		35.43
*Planococcus glaciei* PCL1	Chromosome	SAMN60519689/SRX33759953	CM176595.1	136,628/11,982	401	3,998,358	3,918	46.51
	pPCL1_72		JBYTNU010000002.1			72,286		38.1
	pPCL1_9		JBYTNU010000003.1			9,038		39.88

^*a*^ Total CDS as annotated by NCBI PGAP v6.11; values for PBL6 and PCL1 represent the combined count across all replicons.

Annotation revealed a complete urease operon (*ureABCDEFG*) in LBL1, consistent with its ureolytic phenotype. Interestingly, PBL6 encoded *ureABC* and *ureEFG*, but lacked *ureD*, which is essential for urease activation. β- and γ-class carbonic anhydrase (CA) genes were identified in all three genomes, highlighting their potential role in CA-mediated MICP.

## Data Availability

The complete genome sequences for *Lysinibacillus fusiformis* strain LBL1 (CM176597.1), *Peribacillus frigoritolerans* PBL6 (CM176596.1; pPBL6_12 JBYTNV010000002.1), and *Planococcus glaciei* PCL1 (CM176595.1; pPCL1_72 JBYTNU010000002.1; and pPCL1_9 JBYTNU010000003.1) are all available in the NCBI database under BioProject accession number PRJNA1472847. Raw CCS reads have been deposited in the NCBI Sequence Read Archive (SRA) under accession numbers SRX33759951 (LBL1), SRX33759952 (PBL6), and SRX33759953 (PCL1).
